# Supplementary material for: Modeling influenza transmission dynamics with media coverage data of the 2009 H1N1 outbreak in Korea
Source: PLoS One. 2020 Jun 11;15(6):e0232580. doi: 10.1371/journal.pone.0232580 (PMC7289370; doi:10.1371/journal.pone.0232580)
Supplement: Attachment — Submitted filename: ResponsestoReviewers.pdf [file pone.0232580.s002.pdf]

PONE-D-19-24877
Modeling influenza transmission dynamics with media coverage data of the 2009 H1N1 outbreak in Korea
PLOS ONE

We are grateful for the valuable comments from the two Reviewers. In this revision, we addressed all of the Reviewers' comments and have made an effort to better explain the relevance of our results. We have also updated references and some numerical simulations as recommended by the Reviewers.

Reviewer #1:

Please rewrite the abstract entirely. Most of what is contained in there at the moment is part of the introduction. The abstract should describe the salient points of the work you did and not the history of modeling.

Thanks for the valuable comments. We have rewritten our abstract in the revised manuscript.

Abstract

Recurrent outbreaks of the influenza virus continue to pose a serious health threat all over the world. The role of mass media becomes increasingly important in modeling infectious disease transmission dynamics since it can provide public health information that influences risk perception and health behaviors. Motivated by the recent 2009 H1N1 influenza pandemic outbreak in South Korea, a mathematical model has been developed. In this work, a previous influenza transmission model is modified by incorporating two distinct media effect terms in the transmission rate function; (1) a theory-based media effect term is defined as a function of the number of infected people and its rage of change and (2) a data-based media effect term employs the real-world media coverage data during the same period of the 2009 influenza outbreak. The transmission rate and the media parameters are estimated through the least-squares fitting of the influenza model with two media effect terms to the 2009 H1N1 cumulative number of confirmed cases. The impacts of media effect terms are investigated in terms of incidence and cumulative incidence. Our results highlight that the theory-based and data-based media effect terms have almost the same influence on the influenza dynamics under the parameters obtained in this study. Numerical simulations suggest that the media can have a positive influence on influenza dynamics; more media coverage leads to a reduced peak size and final epidemic size of influenza.

Reviewer #2: Modeling influenza transmission dynamics with media coverage data

The article propose two models for the dynamics of H1N1 influenza at the human population level with different approximations for the mass media M(t). The first approximation is called the theory-based and the second one incorporates a data-based media effect term. The second approach employs data from the 2009 H1N1 incidence in South Korea as well as the real-world media coverage data in the same period.

The authors computed the basic reproduction number R0 without treatment and hospitalization. The numerical results show that the model with a theory-based media effect term and the one with a data-based media effect term produce similar outputs for a moderate value of R0.

In my opinion, the topic is interesting and useful. However, some details need to be addressed to give more clarity and robustness to the results.

• Page 2. “Xiao et al. [11] made a more realistic assumption that people are sensitive not only to the number of infected people, but also to whether the situation is getting better or worse. Thus, they built a model where the transmission rate is a function of both the number of infected people and its rate of change.” Explain better why this considered the theory-based model (page 4). Notice, that a and b are unknowns.

Thanks for the comments. As the reviewer pointed out, there are various modeling approaches on how to incorporate media effects into mathematical models (as given in the introduction of our manuscript pp.2-3). Mathematical models are developed by incorporating the media impact, assuming that this impact is positive, which implies that larger media coverage would reduce the transmission rate. However, most existing models consider only the number of infected individuals in the media effect, while the work [Xiao et al. and Tchuenche et al.] incorporates both the number of infected individuals and its rate of change in their media functions. We have agreed that it would be more realistic to assume that transmission dynamics are influenced by not only the number of infected individuals but also the rate of changes since people tend to pay attention both to the current situation of influenza and whether it is getting better or worse. In addition, as the reviewer pointed out, a and b are unknown in the media term and they are scale constants that represent their relative strength; these parameter values can be estimated to the real outbreak data as we have done in our work. We have updated this issue in the revised manuscript (see page 2).

Y. Xiao and T. Zhao, Dynamics of an infectious disease with media/psychology induced non-smooth incidence.

J.M. Tchuenche and C.T.Tanuch, Dynamics of an Infectious Disease Where Media Coverage Influences Transmission.

• Page 5. Nothing is mentioned about the equilibrium points.

Thanks for the comment. We have included the definition of the equilibrium point in the revised manuscript (see page 5).

• Page 5. Some comment from a dynamical point of view would help to understand why the media M(t) (through the parameters of M(t)) do not play any role in the R0. This result seems counterintuitive.

Thanks for raising this issue. We agree with the reviewer that it is important to investigate the impacts of the media M(t) on influenza transmission dynamics. We assume that the population

is completely susceptible at the beginning of the epidemic and the R0 measures the average secondary cases at the beginning of the epidemic. This implies that media coverage is assumed to have no effect at the beginning of the epidemic. Hence, media coverage does not play a role in the basic reproductive number R0. This is consistent with the results of Xiao et al. and Tchuenche et al.; the parameters of M(t)) do not play any role in their R0, but they presented the impacts of M(t) on their transmission dynamics (the peak time and final epidemic size). This issue has been addressed in the revised manuscript (see page 6).

As the reviewer suggested, we have carried out the effects of the media M(t) on influenza transmission dynamics; incidence and cumulative incidence are presented as we vary the intensity of media coverage, c. These new results have been included in the revised manuscript (see Figures 9-10).

• Line 180. The authors mentioned that the most relevant parameters are p and eta. Why? from which analysis ?

Thanks for the comment. As the reviewer mentioned, we have carried out the sensitivity of R0 with varying each parameter and this new result has been included in the revised manuscript (see Figure 2).

In addition, a proportion 0<p<1 of exposed individuals progress to the infectious class I at the rate k while the rest (1-p) progress to the asymptomatic *partially* infectious class A at the same rate k. Here, eta is the relative constant (0<eta<1) that accounts for the reduction in transmissibility for asymptomatic individuals (*partially infectious*). p and eta are associated with asymptomatic individuals and as investigated in "N. H. L. Leung et al 2015 Epidemiology" that these parameters have higher uncertainty (larger variances) than other parameter values.

N. H. L. Leung et al, The fraction of influenza virus infections that are asymptomatic: a systematic review and meta-analysis, Epidemiology, 2015 26(6) 862-872.

Therefore, based on the sensitivity of R0 and the fact that p and eta have higher uncertainty levels, sensitivity results (for p and eta) are further explored. This has been updated in the revised manuscript (see Figure 3).

• Line 217. Please present, a little bit more of detail of the fits. How do you know that you got the global minimum in each of the 4 windows?

Thanks for the valuable comments. As the reviewer pointed out, this is an important issue for the inverse problem associated with parameter estimations in many epidemic models.

Let $y = (y_1, y_2, \cdots, y_M)$ be the number of cumulative cases in days and the influenza model, $\emptyset(\Theta) = (\emptyset_1, \emptyset_2, \cdots, \emptyset_M)$ be defined in the parameter space $D = \mathbb{R}^n$ (n is the number of parameters).

Note that the influenza model $\emptyset : D \rightarrow F$ associates to each parameter vector Θ an observable vector $\emptyset(\Theta)$ where the component represents the number of cumulative infected cases in days, i.e.,$\emptyset_m = C_m$ (m=1,…,M). In practice, the parameters vector is restricted to be on the convex set of *admissible values* $W = \{\Theta \in D \mid lb \leq \Theta \leq ub\}$, in which lb and ub are lower and upper vector bounds for Θ, respectively.

Given an *observation vector* $y \in F$ and a *prediction vector* $\emptyset(\Theta) \in F$, the calibration aims at finding a vector of parameters Θ^* such that $\Theta^* = arg\min_{\Theta \in W} J(\Theta)$

for a cost function, $J(\Theta) = \|y - \emptyset(\Theta)\|^2 = \{\sum_{m=1}^{M} |y_m - \emptyset_m(\Theta)|^2\}$.

This is the *inverse problem* associated to the epidemic model. Generally, it is highly nonlinear, with none or low regularity, multiple solutions (or even none), being very complicated to solve [Aster et al. and Yaman et al.]. The inverse problem seeks to estimate a finite number of parameters on a finite-dimensional space with a nonlinear cost function. Furthermore, Theorem 4.5.1 of Chavent [*Chavent*] showed a proper sense of well-posedness of the inverse problem (existence, uniqueness and local stability). Also, the Trust-Region-Reflective method (TRR) is employed here to numerically approximate a solution for the inverse problem [*Conn et al.*]. Hence, this ensures the global minimum of the parameter estimation under the above assumptions.

For our parameter estimation procedure, the whole time window is divided into four sub-time windows; [t0, tf] = [t0, t1] ∪ [t1, t2] ∪ [t2, t3] ∪ [t3, tf]. Here, the parameter vector Θ_i denotes β, a, b for Model 1 and β and d for Model 2 (Θ_i, where i=1,2,3,4 is used to distinguishing the parameter values for the four windows). Then, the inverse problem is solved in each of the four windows in a sequential manner. First, the inverse problem is solved for Θ_1, then the next inverse problem for Θ_2 is solved using the initial conditions (which are obtained from the model output values at t1 of [t0, t1]). We complete this sequential process for Θ_3 and Θ_4, respectively.

We have updated this issue in the revised manuscript (see page 7).

R. Aster, B. Borchers, and C. Thurber, editors. Parameter Estimation and Inverse Problems. Elsevier, second edition, 2012. ISBN 978-0-128-10092-9.

F. Yaman, V. G. Yakhno, and R. Potthast. A Survey on Inverse Problems for Applied Sciences. Math. Probl. Eng., 2013, 2013. doi: 10.1155/2013/ 976837.

G. Chavent. Nonlinear Least Squares for Inverse Problems: Theoretical Foundations and Step-by-Step Guide for Applications. Springer, 2010. doi: 10.1007/978-90-481-2785-6.

A. R. Conn, N. I.M. Gould, and P. L. Toint, editors. Trust Region Methods. SIAM, 2000.

• Line 275. Sensitivity of final epidemic size is analyzed just based on a graph. I suggest using a metric (maybe with an integral to add cases) so results can be compared objectively.

Thanks for the comments. We have updated the sensitivity of the final epidemic size with incidence and cumulative incidence under Model 1 and Model 2 (see Figures 9-10 in the revised manuscript). In addition, we have included the corresponding metric of the peak size and the final epidemic size in the revised manuscript (see Table 3).

• Line 314. "The theory-based media effect term made the result excessively dependent on the media effect, which is inconsistent with the results of previous research [6, 8]." Here is important that the authors explain why the results are inconsistent and give details.

Thanks for pointing out this issue. We carefully looked at the results in [6, 8] and we have concluded that a direct comparison between their results and ours is not appropriate since their models include vital dynamics while our model does not. Therefore, their models in [6, 8] have both disease-free equilibrium and endemic equilibrium while our model does have only disease-free equilibrium (not endemic equilibrium). Hence, we have deleted that sentence and updated the discussion in the revised manuscript.
