# Supplementary material for: Modeling influenza transmission dynamics with media coverage data of the 2009 H1N1 outbreak in Korea
Source: PLoS One. 2020 Jun 11;15(6):e0232580. doi: 10.1371/journal.pone.0232580 (PMC7289370; doi:10.1371/journal.pone.0232580)
Supplement: Attachment — Submitted filename: ResponsestoReviewers_R1.pdf [file pone.0232580.s003.pdf]

PONE-D-19-24877R1
Modeling influenza transmission dynamics with media coverage data of the 2009 H1N1 outbreak in Korea
PLOS ONE

We are grateful for the valuable comments from the two Reviewers. In this revision, we addressed all of the Reviewers' comments. We have also updated references and some numerical simulations as recommended by the Reviewer.

Reviewer #1: None. All my previous comments have been addressed. I have no other comments at the moment. My recommendation is ACCEPT

Reviewer#2:

Regarding the effect of media varying c the authors improved the paper. I would like to see what happens with all the classes. It can be seen that with a larger value of c the peak and the total number of cases decrease. One way to do that is to show in a table and figure all the sub-populations at different times of the simulation or at least after one or two years.

Thanks for the comments. We have updated the results of other classes for Model 1 and Model 2 under three different values of media coverage (see p.8-9 and Figures 11-12 in the revised manuscript).

Regarding the uniqueness of the solution of the inverse problem, I am not sure (as authors claim in the response) that their method guarantees a unique global solution. In the response, the authors cited a Theorem (4.5.1) by Chavent. I do not have access (and time) to the Theorem to be able to check if the conditions of the Theorem are satisfied. However, I think the authors can just extend their explanation that they gave in response letter and add the references. In that, way the authors are adding technical details (clarity) to the article as request by this journal. Notice, that the initial question is related to global uniqueness and not the local one. If authors add the paragraph to this point, they will add some strong argument to their paper.

Thanks for the comments. As the reviewer suggested, we have addressed this issue in the revised manuscript (see p. 7-8). The references below have been included in the revised manuscript as well.

R. Aster, B. Borchers, and C. Thurber, editors. Parameter Estimation and Inverse Problems. Elsevier, second edition, 2012. ISBN 978-0-128-10092-9.

F. Yaman, V. G. Yakhno, and R. Potthast. A Survey on Inverse Problems for Applied Sciences. Math. Probl. Eng., 2013, 2013. doi: 10.1155/2013/ 976837.

G. Chavent. Nonlinear Least Squares for Inverse Problems: Theoretical Foundations and Step-by-Step Guide for Applications. Springer, 2010. doi: 10.1007/978-90-481-2785-6.

A. R. Conn, N. I.M. Gould, and P. L. Toint, editors. Trust Region Methods. SIAM, 2000.

Eber Dantas, Michel Tosin, Americo Cunha Jr Calibration of a SEIR–SEI epidemic model to describe the Zika virus outbreak in Applied Mathematics and Computation. 338 (2018) 249–259.*
