# Supplementary material for: Modeling influenza transmission dynamics with media coverage data of the 2009 H1N1 outbreak in Korea
Source: PLoS One. 2020 Jun 11;15(6):e0232580. doi: 10.1371/journal.pone.0232580 (PMC7289370; doi:10.1371/journal.pone.0232580)
Supplement: Attachment — Submitted filename: ResponsetoReviewer_R2.docx [file pone.0232580.s004.docx]

PONE-D-19-24877R2
Modeling influenza transmission dynamics with media coverage data of the 2009 H1N1 outbreak in Korea
PLOS ONE

**We are grateful for the valuable comments from the Reviewer. In this revision, we addressed all of the Reviewers’ comments. We have updated numerical simulations as recommended by the Reviewer.**

Reviewer #2: The authors improved the paper. As I mentioned initially the article is interesting and useful. I like the idea. In the previous review, I suggested to add a figure with all the populations. The authors added Figs 11-12, but without the Susceptible and Vaccinated classes. This is a small thing to add.

**Thanks for the comments. We have updated the results of all classes for Model 1 and Model 2 under three different values of media coverage (see pp.20-21 Figures 11-12 in the revised manuscript).**
